# Draft Genome Sequence of Limisphaera ngatamarikiensis NGM72.4^T^, a Moderately Alkaliphilic Thermophile Belonging to the Class *Verrucomicrobiae*

**DOI:** 10.1128/MRA.00225-20

**Published:** 2020-04-30

**Authors:** Carlo R. Carere, Jason A. Steen, Philip Hugenholtz, Matthew B. Stott

**Affiliations:** a Department of Chemical and Process Engineering, University of Canterbury, Christchurch, New Zealand; b Australian Centre for Ecogenomics, School of Chemistry and Molecular Biosciences, The University of Queensland, St. Lucia, QLD, Australia; c Institute for Molecular Bioscience, The University of Queensland, St. Lucia, QLD, Australia; d School of Biological Sciences, University of Canterbury, Christchurch, New Zealand; Portland State University

## Abstract

Limisphaera ngatamarikiensis NGM72.4^T^ is a thermophilic representative of the class *Verrucomicrobiae*. Isolated from geothermally heated subaqueous clay sediments from a Ngatamariki hotspring in Aotearoa New Zealand, the 3,908,748-bp genome was sequenced using the Illumina HiSeq 2500 platform. Annotation revealed 3,083 coding sequences, including 3,031 proteins, 3 rRNA genes, and 46 tRNA genes.

## ANNOUNCEMENT

Limisphaera ngatamarikiensis NGM72.4^T^ is a Gram-negative, moderately alkaliphilic (optimal pH, 8.1 to 8.4) thermophile (growth optimal at 60 to 65°C) (1). The phylogenetic position of strain NGM72.4^T^, according to GTDB-Tk (2), is a novel member of the class *Verrucomicrobiae* most closely related to the order *Pedosphaerales*, likely representing at least a novel family. This distinguishes it from other described species within the phylum *Verrucomicrobia* (3) and, along with its thermophilic phenotype, makes it of considerable research interest. Here, we report the draft genome sequence of NGM72.4^T^ (1).

The draft genome was generated using genomic DNA isolated from a single colony grown on a basal nutrient salt medium supplemented with mannose (0.5 g liter^−1^) (1) and extracted using a NucleoSpin tissue kit (Macherey-Nagel) per the manufacturer’s instructions. DNA libraries were prepared according to the manufacturer’s protocol using a Nextera DNA library preparation kit (Illumina catalog number FC-121-1031). Upon completion, the quality of each library was checked using an Agilent 2100 Bioanalyzer (Agilent catalog number G2939BA), pooled at 1/40th of a HiSeq 2500 lane, and sequenced at the Institute for Molecular Biosciences at the University of Queensland. Genome sequencing was undertaken using the Illumina HiSeq 2500 platform with the TruSeq sequencing by synthesis (SBS) kit v3-HS (200 cycles) 2 × 100-bp paired-end chemistry according to the manufacturer’s protocol. High-quality reads were generated by clipping adaptors using SeqPrep v1.1 (https://github.com/jstjohn/SeqPrep) and removing low-quality bases using Nesoni v0.108 (https://github.com/Victorian-Bioinformatics-Consortium/nesoni). All parameters were set to default with the exception of –homopolymers = yes, –length = 30, and quality = 15. Velvet v1.2.07 was used through the VelvetOptimiser script v2.2.5 (http://www.vicbioinformatics.com/software.velvetoptimiser.shtml) with the parameters –s = 45, –e = 95, and –x = 4. The draft genome comprises 96 scaffolds from 3,818,865 reads with a mean coverage of 50× and an *N*_50_ value of 114,780 bp. Gene prediction was performed using Prodigal v2.5 (4) with default settings. Annotation was performed using the NCBI Prokaryotic Genome Annotation Pipeline (5). The size of the assembled draft genome is 3,908,748 bp, and the GC content is 64.84%. Annotation revealed 3,083 coding sequences, including 3,031 proteins, 3 rRNA genes, and 46 tRNA genes.

Analysis of the Limisphaera ngatamarikiensis NGM72.4^T^ genome supports observations of an aerobic chemoheterotrophic lifestyle, capable of growth on a variety of simple sugars and sugar derivatives (1). In addition to complete pathways for the oxidative catabolism of carbohydrates via glycolysis, genes encoding the tricarboxylic acid (TCA) cycle and respiratory complexes I through IV were also identified. The oxidative decarboxylation of pyruvate (to acetyl-coenzyme A [acetyl-CoA]) is catalyzed via pyruvate dehydrogenase. Consistent with observations of motility, a full complement of genes encoding flagellar biosynthesis was identified. Despite the abundance and distribution of *Verrucomicrobia* sequence data in environmental surveys, few extant representatives of this phylum are available for study (6, 7). We hope that this genome serves as a useful resource to those interested in isolating other verrucomicrobial strains.

### Data availability.

The whole-genome shotgun sequence of Limisphaera ngatamarikiensis NGM72.4^T^ (1) has been deposited at DDBJ/ENA/GenBank under the accession number JAAKYA000000000. The version described here is the first version, JAAKYA010000000. The sequencing reads are available under BioProject accession number PRJNA606647.
